# Editorial: Heart valve diseases: from molecular mechanisms to clinical implications

**DOI:** 10.3389/fmmed.2023.1260912

**Published:** 2023-07-28

**Authors:** Jaime Ibarrola, Natalia Lopez-Andres

**Affiliations:** ^1^ Molecular Cardiology Research Institute, Tufts Medical Center, Boston, MA, United States; ^2^ Cardiovascular Translational Research, Navarrabiomed, Hospital Universitario de Navarra (HUN), Universidad Pública de Navarra (UPNA), Instituto de Investigacion Sanitaria de Navarra (IdiSNA), Pamplona, Spain

**Keywords:** heart valve disease (HVD), serotonin receptor, mechanical valve, biological valve prosthesis, fibrosis

Heart valve disease (HVD) is considered a serious global health problem. Around 2.5% of the population present some degree of HVD (Aluru et al., 2022) being aortic stenosis (AS) and mitral valve diseases (MVD) the most common forms of HVD (Hollenberg, 2017). This Research Topic “*Heart valve diseases: from molecular mechanisms to clinical implications*” includes five articles aiming at characterizing the molecular mechanisms underlying HVD.

Serotonin or 5-hydroxytryptamine (5-HT) is a multifaceted neurotransmitter that regulates important physiological processes such as cognition or memory and also exerts hormonal, autocrine, paracrine, and endocrine actions (Young, 2007). Soluble serotonin can bind to 5-HT receptors: 2A and 2B receptors and activates the Erk1/2 signaling pathway. Erk1/2 pathway is involved in cell proliferation, cell differentiation, and extracellular matrix (ECM) remodeling (Liu et al., 2019). In the past decades, several authors showed that serotonin pathway plays a role in the development of HVD. Thus, the overactivation of 5-HT2A-2B receptors by 5-HT agonist drugs (Ergot derivates, dopamine agonists, amphetamine derivates) can contribute to the development of HVD through valve interstitial cells (VICs) activation and proliferation (Jian et al., 2002; Elangbam, 2010). Most of the studies have been performed in the context of MVD (Thalji et al., 2015; Ayme-Dietrich et al., 2017; Driesbaugh et al., 2018; Ayme-Dietrich et al., 2019; Ibarrola et al., 2020; Garcia-Pena et al., 2021), showing that both receptors are overactivated in mitral valve prolapse.

In this Research Topic, the review by Waldum and Wahba described the mechanisms of serotonin signaling in HVD development more in detail. Serotonin is transported in the blood by platelets. The authors suggested that in HVD, i.e., AS, platelets can release serotonin directly in the valves inducing HVD through 5-HT2B receptor activation. Waldum and Wahba summarize the main studies that demonstrate the involvement of serotonin receptors in HVD using different animal models (Sheep, mouse, and rat) as well as human samples. Otherwise, no clinical studies have been conducted to analyze the impact of 5HT receptor expression or the impact of the 5HT receptor antagonist in humans.

One original research article included in this Research Topic (Schussler et al.) quantified for the first time the most frequent 5-HT receptors in normal human valves (aortic, pulmonary, mitral, and tricuspid valves) without disease. The authors demonstrated the presence of 5-HT2A and 5-HT2B receptors in human valves. Moreover, they described that the expression of both receptors is similar, and they did not observe differences between the right and left side valves. The authors suggest that these receptors play an important role in the heart valves. The expression of 5-HT4 receptor was high in human valves compared with 5-HT1 receptors and in the same quantity as the 5-HT2A receptor and 5-HT2B receptor. Of note, the expression of 5-HT4 receptors has been classically studied in the myocardium, being this study the first that analyzes its expression in healthy human valves. Finally, this work showed some positive correlations between 5-HT1A receptor and 5-HT1B/D receptor, and between 5-HT4 receptor and 5-HT7 receptor. The authors propose that these correlations can be associated with homodimerization or heterodimerization of the receptors. However, further studies are needed to confirm the interaction between the different 5-HT receptors.

Currently, no pharmacological treatment has been shown to prevent HVD development or progression. According to the current guidelines, medical treatment is reserved to palliate the symptoms caused by HVD, such as diuretics for congestion or fluid retention or drugs to slow heart rate if arrhythmias occur. The current management is to follow the patients until symptoms appear, or serial image testing shows an impact on cardiac structure and function; in any of these situations, a mechanical procedure on the valve is indicated (Maganti et al., 2010; Zoghbi et al., 2017; Baumgartner et al., 2018). The standard treatment is surgical intervention, performing replacement of the diseased valve with a prosthetic valve (mechanical or biological valves) mimicking native biomechanical function or valve repair if anatomically feasible. Mechanical valves are made of synthetic materials, whereas biological heart valves are made of tissues and valves derived from animals or human donors. As a part of the post-surgery treatment, the mechanical or biological prosthesis valve must be analyzed to confirm the correct function, usually using echocardiography (Sigala et al., 2023).

A brief research report written by Derda et al. proposed new studies for mechanical valve analysis. Cinefluoroscopy (CF) is a non-invasive method to measure leaflet function. CF allows exact measurements of opening and closing angles of mechanical heart valve leaflets. CF is a complementary method of echocardiography. Studies on the application of CF are lacking and guidelines for the correct measurements are not defined. 94 patients with 118 CF studies performed by 31 different investigators were included in the study. CF studies were divided into three groups based on the visualization quality: sufficient, suboptimal or unsuitable. This study is the first that investigate the imaging quality of mitral valves by CF. Only 25% of the CF studies were sufficient for a correct evaluation of the mitral valve function. The results conclude that there are not enough good CF studies due to the lack of knowledge and education.

Biological valves usually are made with bovine or porcine tissues. The big issue of the biological valves is their durability, including the presence of residual free aldehyde groups, toxicity, and calcification. These free aldehyde groups can induce calcification and reduce the proteoglycans content in the valves contributing to reducing the durability of biological valves (Head et al., 2017). Liu et al. in the original research article studied how to neutralize the free aldehyde groups with adipic dyhydrazide (ADH) and provide sites to bind with oligohyaluronan (OHA) to increase the content of glycosaminoglycans in the biological valve. The authors demonstrated that ADH significantly reduces the residual free aldehyde groups in bovine pericardial tissue. Furthermore, they showed that calcification and inflammation are reduced in pericardial tissue from rat subcutaneous implantation model. Finally, they concluded that blocking free aldehydes with ADH and loading OHA improved biocompatibility and toxicity properties. This strategy may be a promising candidate for the next-generation of biological valves.

HVD can lead to the development heart failure, as a consequence of the heart cannot pump blood correctly. The manuscript by Lunde et al. investigated the Calcineurin-NFAT pathway on myocardial remodeling associated with AS. They performed a translational study using myocardial biopsies from AS patients as well as a mouse model of pressure overload that exhibits cardiac hypertrophy (aortic banding). The authors demonstrated the dynamic activation of the Calcineurin-NFAT signaling pathway. They showed an increase in NFATc1-4 proteins in AS patients and in the acute phase of pressure overload in the mouse model. Furthermore, the authors demonstrated that NFAT activation is reversible when the pressure overloud is reversed. They conclude that Calcineurin-NFATc activation is a sustained response to pressure overload and the attenuation of the NFATc pathway is important for reverse remodeling and outcome after aortic valve replacement in AS patients.

Overall, this Research Topic covers several studies on HVD, including new pathways involved in HVD development, new strategies for follow-up patients after valve replacement, and secondary disease associated with HVD, offering insight into the challenging problem of treatment HVDs.
